# Spatial database of planted forests in East Asia

**DOI:** 10.1038/s41597-023-02383-w

**Published:** 2023-07-22

**Authors:** Akane O. Abbasi, Xiaolu Tang, Nancy L. Harris, Elizabeth D. Goldman, Javier G. P. Gamarra, Martin Herold, Hyun Seok Kim, Weixue Luo, Carlos Alberto Silva, Nadezhda M. Tchebakova, Ankita Mitra, Yelena Finegold, Mohammad Reza Jahanshahi, Cesar Ivan Alvarez, Tae Kyung Kim, Daun Ryu, Jingjing Liang

**Affiliations:** 1 Forest Advanced Computing and Artificial Intelligence (FACAI) Lab, Department of Forestry and Natural Resources, Purdue University, 715 W State St., West Lafayette, IN 47907 USA; 2 State Key Laboratory of Geohazard Prevention and Geoenvironment Protection, Chengdu University of Technology, Dongsanlu, Erxianqiao, Chengdu, 610059 Sichuan P.R. China; 3 College of Ecology and Environment, Chengdu University of Technology, Dongsanlu, Erxianqiao, Chengdu, 610059 Sichuan P.R. China; 4 World Resources Institute, 10 G Street N.E., Washington, DC 20002 USA; 5 National Forest Monitoring (NFM) Team, Forestry Division, Food and Agriculture Organization of the United Nations, Viale delle Terme di Caracalla, 00153 Rome, Italy; 6 Helmholtz Center Potsdam GFZ German Research Centre for Geosciences, Section 1.4 Remote Sensing and Geoinformatics, Telegrafenberg, Potsdam, 14473 Germany; 7 Department of Agriculture, Forestry, and Bioresources, Seoul National University, Seoul, 08826 Republic of Korea; 8 Research Institute of Agriculture and Life Sciences, Seoul National University Seoul, Seoul, Republic of Korea; 9 Interdisciplinary Program in Agricultural and Forest Meteorology, Seoul National University, Seoul, 08826 Republic of Korea; 10 National Center for AgroMeteorology, Seoul, Republic of Korea; 11 Research Center of Forest Management Engineering of State Forestry and Grassland Administration, Beijing Forestry University, Beijing, 100107 P.R. China; 12 Forest Biometrics, Remote Sensing and Artificial Intelligence Lab (Silvalab), School of Forest, Fisheries, and Geomatics Sciences, University of Florida, 342 Newins-Ziegler Hall, Gainesville, FL 32611 USA; 13 Sukachev Forest Institute, FRC KNC, Siberian Branch, Russian Academy of Sciences, Academgorodok, 50/28, Krasnoyarsk, 660036 Russia; 14 Lyles School of Civil Engineering, Purdue University, 550 Stadium Mall Drive, West Lafayette, IN 47907 USA; 15 Elmore Family School of Electrical and Computer Engineering, Purdue University, 465 Northwestern Ave, West Lafayette, IN 47907 USA; 16 Environmental Research Group for Sustainable Development (GIADES), Salesian Polytechnic University, Rumichaca y Moran Valverde, Quito, EC 170702 Ecuador; 17 Urban Forests Division, National Institute of Forest Science, Seoul, 02455 Republic of Korea

**Keywords:** Forestry, Forestry, Forest ecology

## Abstract

Planted forests are critical to climate change mitigation and constitute a major supplier of timber/non-timber products and other ecosystem services. Globally, approximately 36% of planted forest area is located in East Asia. However, reliable records of the geographic distribution and tree species composition of these planted forests remain very limited. Here, based on extensive *in situ* and remote sensing data, as well as an ensemble modeling approach, we present the first spatial database of planted forests for East Asia, which consists of maps of the geographic distribution of planted forests and associated dominant tree genera. Of the predicted planted forest areas in East Asia (948,863 km^2^), China contributed 87%, most of which is located in the lowland tropical/subtropical regions, and Sichuan Basin. With 95% accuracy and an F1 score of 0.77, our spatially-continuous maps of planted forests enable accurate quantification of the role of planted forests in climate change mitigation. Our findings inform effective decision-making in forest conservation, management, and global restoration projects.

## Background & Summary

Planted forests are forest ecosystems established by artificial tree planting or seeding for the provision of income and goods, as well as for climate change mitigation and the restoration of ecosystem services and processes^1,2^. According to the Food and Agriculture Organization of the United Nations (FAO)^2^, planted forests globally increased by 41,000 km^2^ per year between 2000 and 2020 and currently amounted to approximately 2,930,000 km^2^. Today, FAO estimates that 36% of the world’s planted forests are distributed in East Asian countries, namely China, Japan, the Republic of Korea (ROK), and the Democratic People’s Republic of Korea (DPRK)^2^. In East Asia, a large proportion of forest area is planted forests (39% in China, 41% in Japan, 36% in ROK, and 16% in DPRK in 2020, according to FAO^2^), while other regions in the world remain well below 20% (19% in Africa, 7% in Europe, and 9% in the United States). Unlike Western countries, where planting was traditionally conducted for silvicultural practices, East Asian countries planted trees for varying purposes with local species and unique history^3–11^.

East Asian countries have implemented a variety of tree-planting policies at different spatial and temporal scales. China leads all countries worldwide with the largest estimated plantation area of about 840,000 km^2^. Since the end of the 1970s, China has established several afforestation projects, including the Three-North Forest Shelterbelt Program^4^, the Natural Forest Conservation Program (also known as Natural Forest Protection Program), and the Grain to Green Program (GGP; also known as the Sloping Land Conversion Program)^5,6^. Currently, China has committed to preserving and expanding forest cover, aiming at mitigating soil erosion, air pollution, and climate change in the coming decades^7^. Although hundreds of tree species have been used for plantation establishment in China, a few species dominate the planted forests across the country, such as Chinese fir (*Cunninghamia lanceolata*) and eucalyptus (*Eucalyptus* spp.)^8^. In Japan, most planted forests were established after World War II to meet the growing demand for timber and other wood products. Thus, fast-growing and highly productive species, such as Japanese cedar (*Cryptomeria japonica*) and Hinoki cypress (*Chamaecyparis obtusa*), were extensively planted^9^. ROK underwent severe deforestation and forest degradation during World War II and the Korean War (1950–1953), followed by active conversion of forests to agricultural lands due to post-war poverty^10^. In response, the government implemented five National Forest Development Plans from 1973 to 2017. A variety of fast-growing species were planted during this period, and the successful recovery of healthy forests and ROK’s sustainable management strategies are internationally recognized^10,11^.

With active tree planting being implemented throughout the world for climate change mitigation, forest restoration, and biological conservation, it has become urgent to establish cost-effective guidelines for all ongoing and upcoming tree-planting projects. Assessment of the costs and benefits of planted forests, the key to the development of such cost-effective guidelines, is contingent on knowing where the existing planted forests are distributed^12–16^ and which tree species are planted^17^. The geospatial distribution of planted forests in East Asia still remains unclear due to a scarcity of complete, transparent, and publicly accessible data records. National governments have published some planted forest maps based on site visits, forest inventory, and satellite data. Yet, the spatial coverage is incomplete for Japan^12^, and the map produced by the Chinese Forest Inventory remains unverified and largely inconsistent with independent studies^13,14^. The existing large-scale maps of planted forests are based on inconsistent data sources with varying reliability and scale^13^ or solely based on satellite images^14^. Because of these differences in spatial extent, underlying data sources, and methods in existing datasets, a database that provides complete, consistent, and ground-truth-based records of the geographic distribution of planted forests and associated dominant tree species for East Asia constitutes a consistent and harmonized product.

Here, we produced the spatial database of planted forests in East Asia at a 1-km resolution and identify dominant tree species in these planted forests to the genus level. Our planted forest map encompasses forests of all ages planted for various purposes, including forest restoration, commercial plantation, and disaster prevention. These mapping products are based on ensemble machine learning models, data fusion, and multi-source data of planted forests. Our multi-source data comprised ~7,000 ground-truth inventory plots in China, five independent digitized maps across the study region, as well as 57 auxiliary datasets and layers, including satellite data such as the Global Ecosystem Dynamics Investigation (GEDI)^18^ and Moderate Resolution Imaging Spectroradiometer (MODIS) data to account for potential differences in forest structure and vegetation characteristics between planted and natural forests. In addition to the main products, we also estimated the upper and lower bounds of potential planted forest extent to account for the uncertainty associated with the varied quality of multi-source training data. With previous records of planted forests being inconsistent in resolution, quality, and accessibility, our map provides a complete, consistent, and *in situ* data-based estimation of the extent and species distribution of planted forests in East Asia.

## Methods

**Fig. 1:**
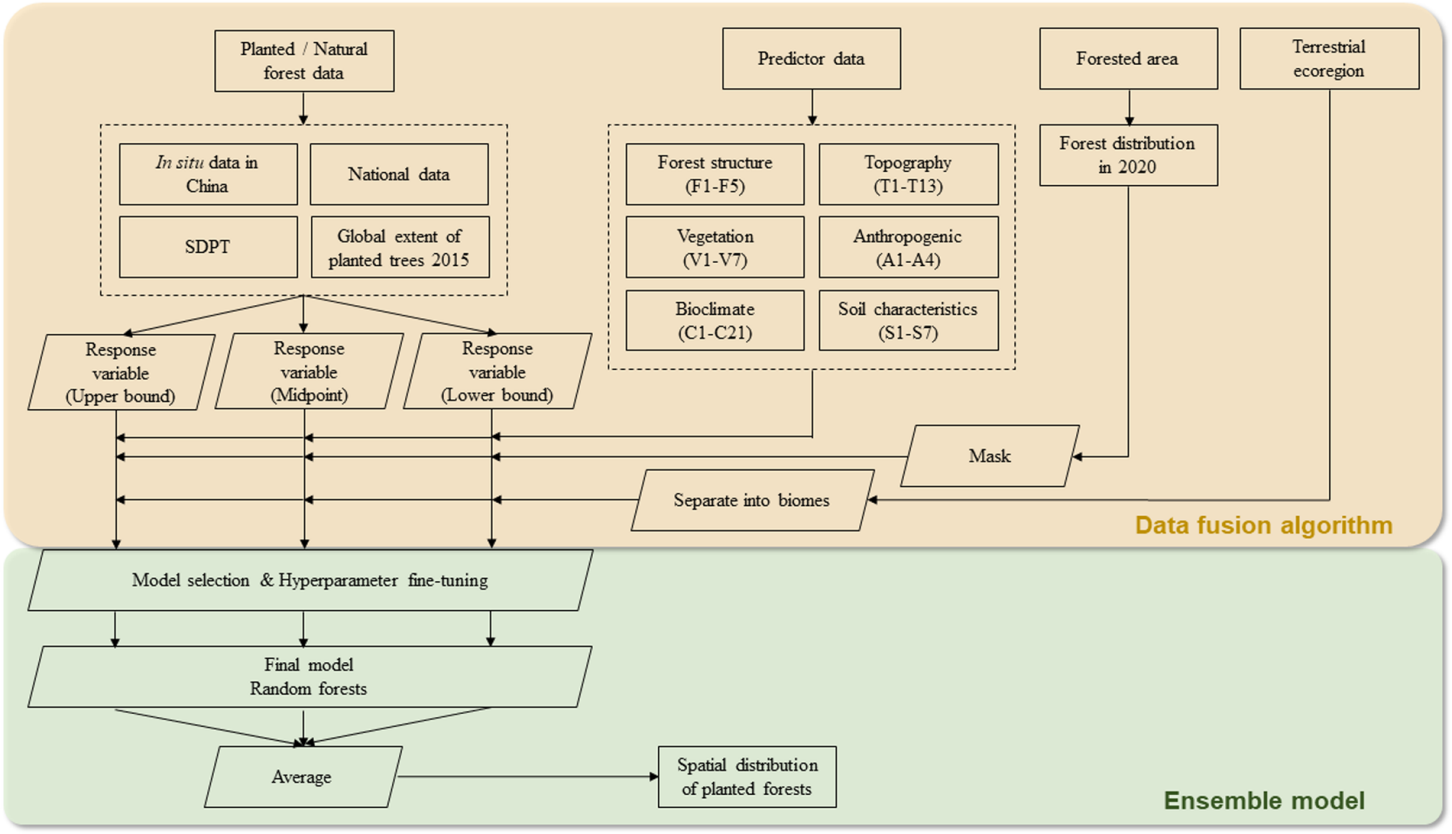
Workflow for developing the spatial database of planted forests. The top section (yellow) represents the data fusion algorithm we used to integrate multi-source data into coherent training datasets. The bottom section (green) represents the ensemble model we developed to predict the spatial patterns of planted forests.

To estimate the spatial distribution of planted forests over East Asia, we integrated multi-source planted-natural forest data from multiple *in situ* inventories and digitized data sources in a high-level data fusion algorithm (Fig. 1). For each observation, we first created a response variable explicitly labeled as either “planted” or “natural” forests. We then obtained data on 57 potential predictor variables encompassing forest structure, vegetation characteristics, bioclimate, topography, anthropogenic information, and soil characteristics, and merged these layers with the response variable layer based on spatial coordinates. The training dataset was then masked to the forested area in 2020 and separated into three biomes based on the Nature Conservancy Terrestrial Ecoregions map^19^. For each biome, we selected the optimal machine learning classification model and fine-tuned hyperparameters. Finally, we mapped planted forest distribution and the distribution of the dominant tree species in these forests to the genus level. Our study area covers China, Japan, ROK, and DPRK.

### Data fusion

**Fig. 2:**
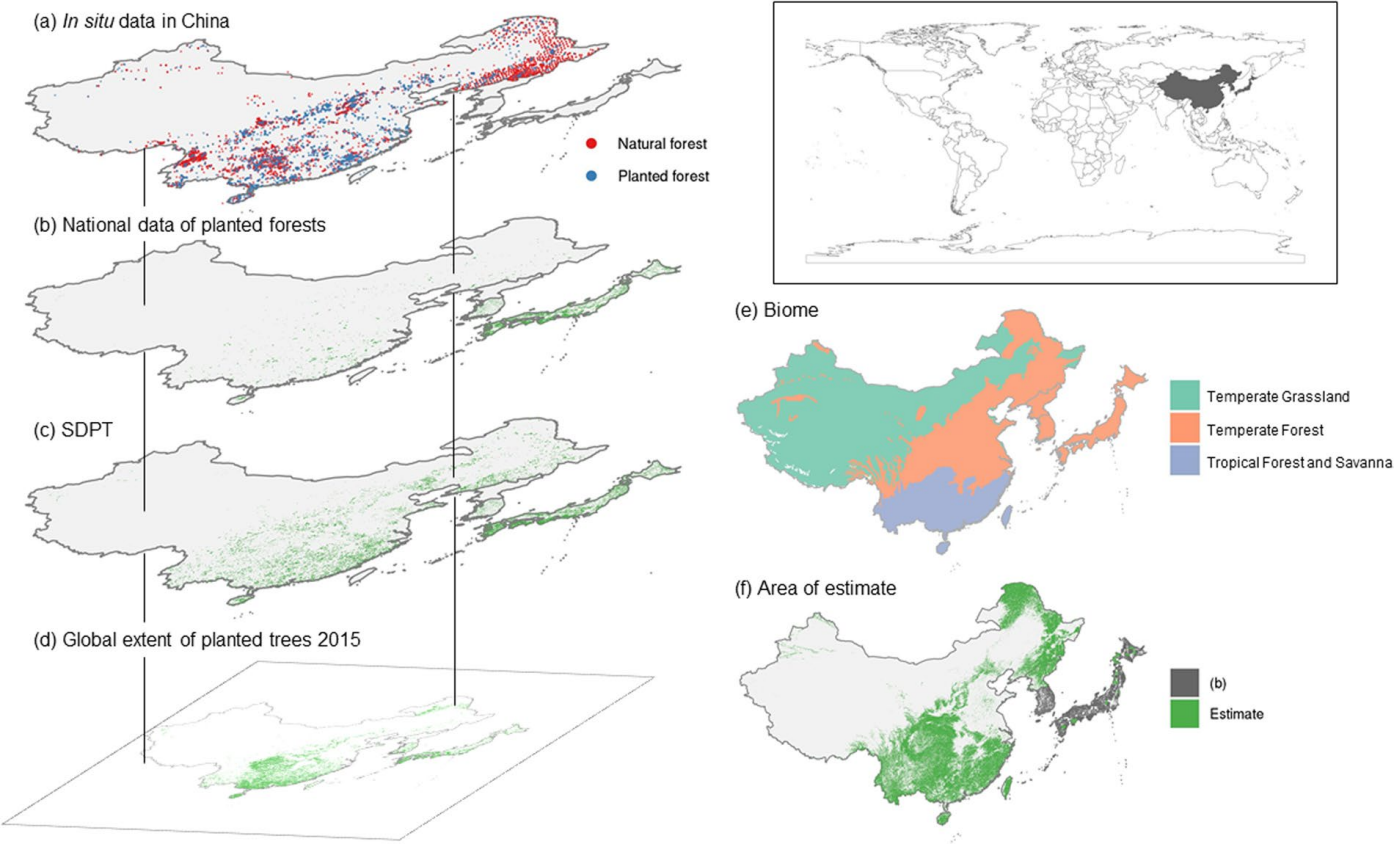
Training data consists of a series of *in situ* and digital maps of planted-natural forest data from multiple independent sources. (**a**) The *in situ* data in China encompass 2,542 and 4,394 ground observations^20–265^, which represent confirmed locations of planted and natural forests, respectively, by previously published articles. (**b**) National maps of planted forests were obtained for China^15^, Japan^12^, and ROK^16^. (**c**) The Spatial Database of Planted Trees (SDPT version 1.0)^13^. (**d**) An estimated Global Extent of Planted Trees 2015^14^. (**e**) Distribution of the three biomes in our study area. We developed a machine learning classification model for each biome to predict planted forests. Note that forests are distributed in the Temperate Grassland according to the FAO’s definition of forest (≥5 m tree height)^2,270^ although the area is limited. (**f**) Distribution of planted forests was estimated mainly for China, DPRK, and small areas in Japan. For the ROK and the majority of Japan, the national planted forest maps^12,16^ (**b**) were used as a final label.

We collected and integrated *in situ* and digitized planted-natural forest data from multiple independent sources using a high-level data fusion algorithm (Fig. 1). Observations from China came from published literature^20–265^ (Fig. 2a), which included 2,542 and 4,394 *in situ* records of confirmed locations of planted and natural forests, respectively. The *in situ* planted forest observations include the plantation of commercial species, such as pine (*Pinus* spp.) and eucalyptus (*Eucalyptus* spp.), and forests planted for restoration purposes. We also obtained the national planted forest map of China (Fig. 2b)^15^, which depicts the distribution of planted forests in 2000. Data specific to Japan was obtained from the national vegetation map created based on site visits and satellite images, where “planted forest” was one of the attributes of vegetation types (Fig. 2b)^12^. This “planted forest” attribute includes restoration-oriented forests composed of broadleaf species, commercial forests dominated by productive species like Japanese cedar (*Cryptomeria japonica*) and Hinoki cypress (*Chamaecyparis obtusa*), and disaster prevention planting, such as Japanese black pine (*Pinus thunbergii*) from coastal erosion and tropical species (*e.g*., *Acacia confusa*) as windbreaks. The national vegetation map has been gradually developed and improved since 2005. Finally, data specific to ROK was a polygon map of planted and natural forests from the national forest cover map (Fig. 2b)^16^. The ROK maps depict the distribution of planted and natural forests from 2009 to 2013, depending on the province. In addition to the country-specific data, we obtained the Spatial Database of Planted Trees covering China, Japan, and ROK (SDPT version 1.0; Fig. 2c)^13^ and a global extent of planted trees 2015^14^, which includes the land use classes of planted forest, woody plantations, and agroforestry of the global forest management map^266^ (Fig. 2d). There is no data specific to DPRK used in this study due to the lack of available data.

To prepare a training dataset for machine learning classification models, we prepared a 0.009° by 0.009° grid (approximately 1 km^2^) for the study region in East Asia. National planted forest maps of China^15^, Japan^12^, and ROK^16^, as well as SDPT^13^ and the Global Extent of Planted Trees^14^ were extracted to the centroid of each grid cell using the “sf” or “raster” packages in R^267,268^. China’s *in situ* observations were associated with each grid cell by taking the majority vote of *in situ* points within each grid cell to determine whether that cell is a planted or natural forest. Grid cells with a 50/50 vote were removed from the training dataset. We then derived the response variable – a label of “planted” or “natural” forest – based on these underlying datasets following the Quality-Oriented Data Integration (QODI).

### Quality-oriented data integration (QODI)

**Fig. 3:**
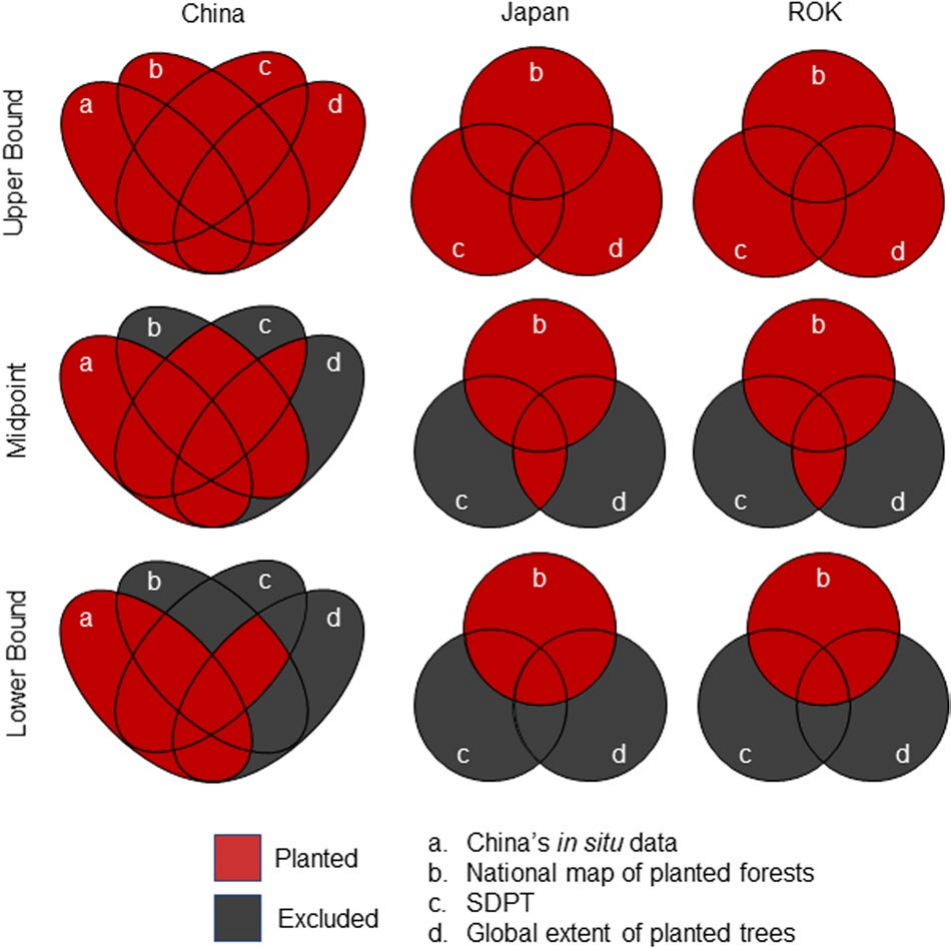
The response variable (“planted” or “natural” forest) was defined in a quality-oriented data integration approach based on multiple underlying data sources. Underlying datasets a-d correspond to Fig. 2a–d. Upper and lower bound models represent the most liberal and conservative approaches in labeling planted forest, respectively. The grey area was removed from the respective training dataset. All areas outside of the Venn diagrams were labeled natural forest. DPRK is not included in this figure due to the absence of training data associated with the country.

Since the underlying datasets differed in data sources and estimation methods, we developed a quality-oriented data integration approach in which the response variable was defined in three different levels of integration (Fig. 3). For each level of integration, we trained a separate set of machine learning models, so that we can quantify the potential range in estimated planted forest areas.

The first level of integration took the most conservative approach in deriving the lower bound of our estimation. Since China’s *in situ* observations^20–265^, Japan’s national vegetation map^12^, and ROK’s national planted forest map^16^ were largely based on *in situ* observations, we labeled a unit forest area (*i.e*., grid cell) as planted if and only if the grid cell was identified as a planted forest by either of these *in situ*-based datasets or identified by at least three other datasets as a planted forest.

The second level of integration took a midway approach in which, in addition to planted forests identified in the first level of integration, a given grid cell was also labeled as a planted forest if two out of the national planted forest maps of China^15^, SDPT^13^, and Global Extent of Planted Trees^14^ datasets agreed so.

The third level of integration took the most liberal approach in deriving the upper bound of our estimation, in which we assumed all underlying data sources were equally reliable and labeled a given grid cell as planted forest if it was identified as a planted forest by either of these datasets.

We also compiled 57 predictor variables for the supervised learning of the classification models (Fig. 1, Supplementary Table S1). The predictor variables consisted of five forest structure attributes^269,270^, seven MODIS-derived vegetation characteristics, 21 bioclimatic attributes^271–274^, 13 topographic attributes^275^, four anthropogenic attributes^276–279^, and seven soil attributes^280^. We obtained four forest structure attributes from the most recent Global Ecosystem Dynamics Investigation (GEDI) dataset, namely canopy height (rh100), plant area index (pai), foliage height diversity (fhd_normal), and total canopy cover (cover) (see Supplementary Table S1)^18,269^. We downloaded the raw footprint-level GEDI data (L2B), among which only full-power lasers were used in this study to ensure the accuracy of the measurement. GEDI data was processed using the “rGEDI” package in R^281^. Another forest structure attribute, tree height^270^, represents the 90^th^ or 95^th^ percentile of energy return height relative to the ground.

We extracted predictor variables to the centroid of each grid cell using the “sf” or “raster” packages in R^267,268^. GEDI footprint-level data was associated with each grid cell by taking the mean value of each attribute. We kept only grid cells with a minimum of 5 m tree height in accordance with FAO’s definition of “forest”^2,270^. Our final training dataset encompassed more than 1.5 million grid cells for the upper bound dataset, 1.0 million grid cells for the midpoint dataset, and 0.9 million grid cells for the lower bound dataset, consisting of one response variable labeled as either “planted” or “natural” and 57 predictor variables. Finally, to account for the differences in terrestrial ecoregions, we divided the overall training dataset into three biomes (Fig. 2e). Based on the global terrestrial biome map^19^, Temperate Grassland/Savanna and Montane and Flooded Grassland were grouped into “Temperate Grassland”. Temperate Broadleaf and Mixed and Temperate Conifer were grouped into “Temperate Forest”, and Tropical Moist, Tropical Dry, and Tropical Grassland/Savanna were grouped into “Tropical Forest and Savanna.” The three biomes remained separated for the upper bound dataset, but Temperate Grassland and Temperate Forest were merged for the midpoint and lower bound datasets to form the “Temperate Forest and Grassland” biome due to low sample size in Temperate Grassland.

For mapping purposes, we prepared another 0.009° by 0.009° grid (approximately 1 km^2^), covering forested area (≥5 m tree height)^2,270^ in the study region with all predictor variables (new data; Fig. 2f). We chose the resolution 0.009° to align with most of the predictor variables (Supplementary Table S1). After a machine learning classification model was trained, estimation was made for each grid cell of this new data. For ROK and a majority of areas in Japan, however, we utilized the existing planted forest maps, namely the national forest cover map of ROK^16^ and the national vegetation map of Japan (Fig. 2b)^12^, respectively, to label the grid cells. Since reliable planted forest data already exist for these areas, we used our estimation only for the remaining areas in China, DPRK, and a small portion of Japan (Fig. 2f). Nevertheless, the existing data for ROK and a majority of areas in Japan were converted to the 0.009° resolution within the forested area for consistency. For the areas where our estimation is used, we imputed missing values in predictor variables of the new data using the “Hmisc” package in R^282^ to provide a spatially continuous map. For the GEDI attributes (Supplementary Table S1), however, we imputed missing values by training random forest (RF) models (see below for details of RF) with seven MODIS attributes due to a large number of missing values (22%, 34%, and 44% of the sample size for the upper bound, midpoint, and lower bound dataset, respectively). For the midpoint and lower bound datasets, we used the average predicted values from 10 repetitions of random forest models using 200,000 data points to minimize computational time (Table 1). To assess the performance of the RF model in imputing missing values in GEDI attributes, we performed cross-validation using bootstrapping. For the upper bound dataset, we randomly sampled the dataset into the training (90%) and testing (10%) sets with replacement. For the midpoint and lower bound datasets, we randomly sampled 200,000 data points for the training sets with replacement, and the remaining was used as the testing dataset (Table 1). Based on 20 random iterations, we calculated the 95% confidence interval (CI) of the root mean square error (RMSE) and R-squared (R^2^). We calculated a 95% CI using the t_0.975_ value with 19 degrees of freedom.

**Table 1:** Summary of tasks conducted in this study.

Model objective	Machine learning	Level of integration	Response	Predictors	Training size	Testing size	Iterations
To impute GEDI attributes in new data for mapping purposes (final model)	Random forests (RF)	Upper Bound	Each GEDI attribute	Seven MODIS attributes	All	NA	1
		Midpoint			200,000 data points	NA	10
		Lower Bound			200,000 data points	NA	10
To impute GEDI attributes in new data for mapping purposes (cross-validation)	RF	Upper Bound	Each GEDI attribute	Seven MODIS attributes	90%	10%	20
		Midpoint			200,000 data points	Remaining points	20
		Lower Bound			200,000 data points	Remaining points	20
To determine the best machine learning model to predict planted forest	RF, support vector machines (SVM), and XGBoost	Upper Bound	Planted or natural	All listed in Supplementary Table S1	50,000	Remaining points	10
		Midpoint			50,000	Remaining points	10
		Lower Bound			80%	20%	10
To fine-tune hyperparameters of the final RF model to predict planted forest	RF	Upper Bound	Planted or natural	All listed in Supplementary Table S1	50,000	Remaining points	10
		Midpoint			50,000	Remaining points	10
		Lower Bound			80%	20%	10
To predict planted forest (final model)	RF	Upper Bound	Planted or natural	All listed in Supplementary Table S1	All	NA	20
		Midpoint			All	NA	20
		Lower Bound			All	NA	20
To predict dominant tree genus (final model)	RF	Upper Bound	Genus	All listed in Supplementary Table S1 except for roadless areas and GEDI	All	NA	1
		Midpoint			All	NA	1
		Lower Bound			All	NA	1
To predict dominant tree genus (cross-validation)	RF	Upper Bound	Genus	All listed in Supplementary Table S1 except for roadless areas and GEDI	90%	10%	100
		Midpoint			90%	10%	100
		Lower Bound			90%	10%	100

### Ensemble machine learning model

We developed an ensemble model to estimate the spatial distribution of planted forests, with three candidate machine learning models: RF, support vector machines (SVM), and XGBoost. RF is a non-parametric ensemble learning approach^283^, which combines a variant of decision trees and an additional level of randomness by bootstrapping sub-data and different sets of predictor variables to mitigate potential multicollinearity issues often encountered in multidimensional machine learning models^284^. We used the “randomForest” package in R^285^. SVM is a supervised learning model which constructs a hyperplane or set of hyperplanes in a high- or infinite-dimensional space to help data analysis^286^. We used the “e1071” package in R^287^. XGBoost is a gradient-boosted decision tree machine learning, designed to accommodate large data at high speed. We used the “xgboost” package in R^288^. The three candidate models are frequently used in ecological and biological research with satisfactory performance^266,289^. Other potential candidate models include artificial neural networks, k-nearest neighbor, Naïve Bayer, *etc*., which are not necessarily superior^290^. All modeling processes were conducted in R^291^.

**Fig. 4:**
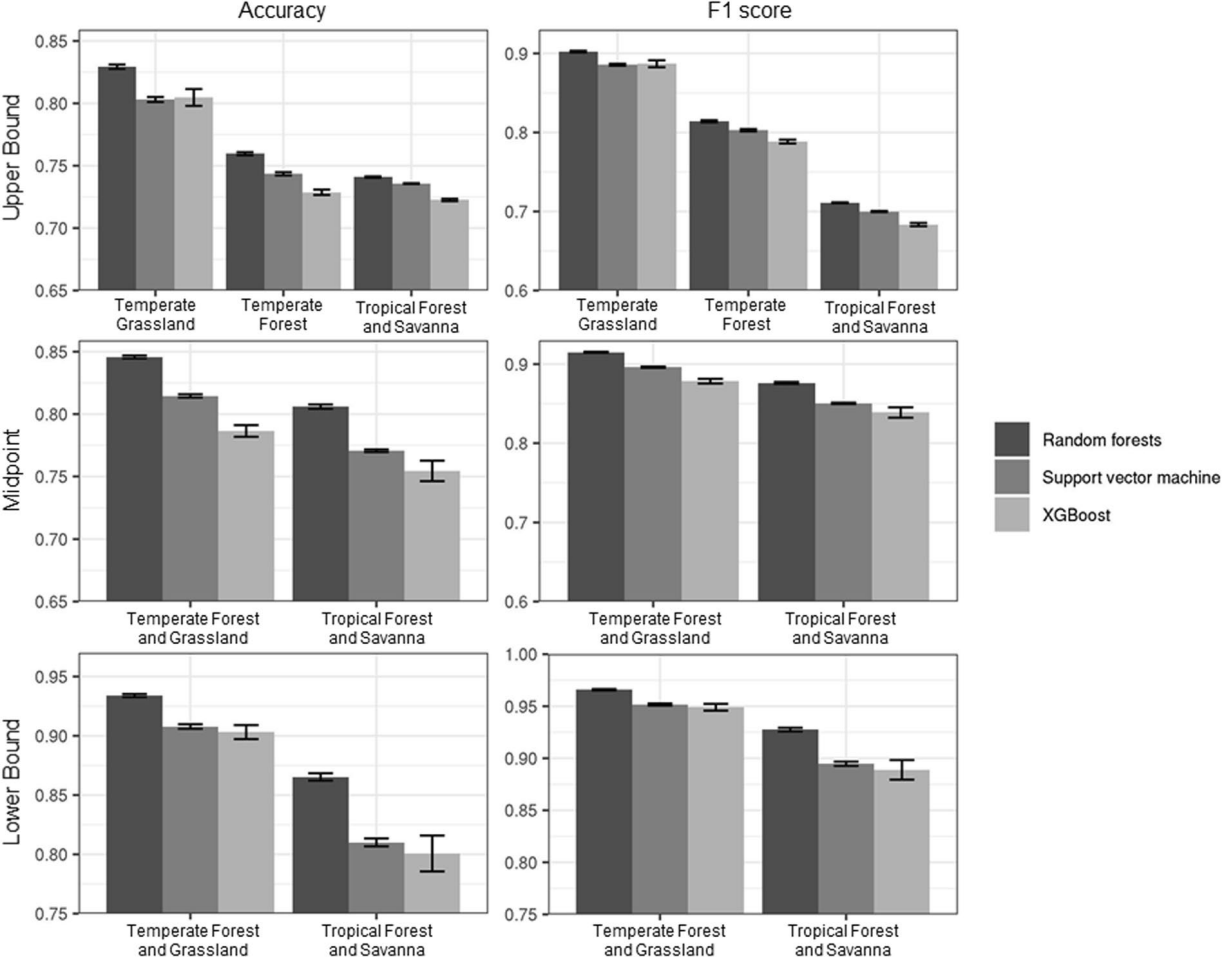
Performance of three candidate machine learning models to map planted forests. Classification accuracy and F1 score of random forest (RF), support vector machine (SVM), and XGBoost imputation models are shown. Mean values from 10 repetitions and 95% confidence intervals are shown for each biome. RF outperformed SVM and XGBoost in all cases, and thus RF was used to model planted forests in our study.

To assess the performance of the three candidate models in estimating planted forests, we conducted cross-validation using bootstrapping. Due to data size, we randomly sampled 50,000 points (25,000 for each class) for the upper bound and midpoint datasets and 80% of the sample points for the lower bound dataset for each of the ten repetitions to create the training set and the rest composed the testing set (Table 1). Default hyperparameter values were used for the three candidate models. Based on 10 iterations, we calculated the 95% CI of classification accuracy and F1 score. We calculated a 95% CI using the t_0.975_ value with 9 degrees of freedom. Classification accuracy shows the proportion of overall correct prediction. While accuracy is the most widely used and intuitive evaluation metric of a classification problem, it overestimates the performance of imbalanced data. F1 score is an equal measure of precision and recall and is more appropriate for imbalanced data^292^. Precision represents the correct prediction of the positive class (*i.e*., planted) among all positive predictions, and recall represents the correct prediction of the positive class among all actual positive cases^293^. Since precision and recall are in an inverse relationship, the combined metric, F1 score, provides a better evaluation perspective of incorrectly predicted cases. Using both accuracy and F1 score, we present a suite of evaluation metrics of our candidate models for both correct and incorrect predictions of an imbalanced dataset. Other potential evaluation metrics include Cohen’s Kappa. However, we did not use it in our study due to the controversy of its use^294^. Compared with SVM and XGBoost, the RF model was 0.7–8.1% more accurate in terms of overall classification accuracy and 1.4–4.5% more reliable in terms of F1 score (Fig. 4). Thus, we chose RF as the final model.

**Fig. 5:**
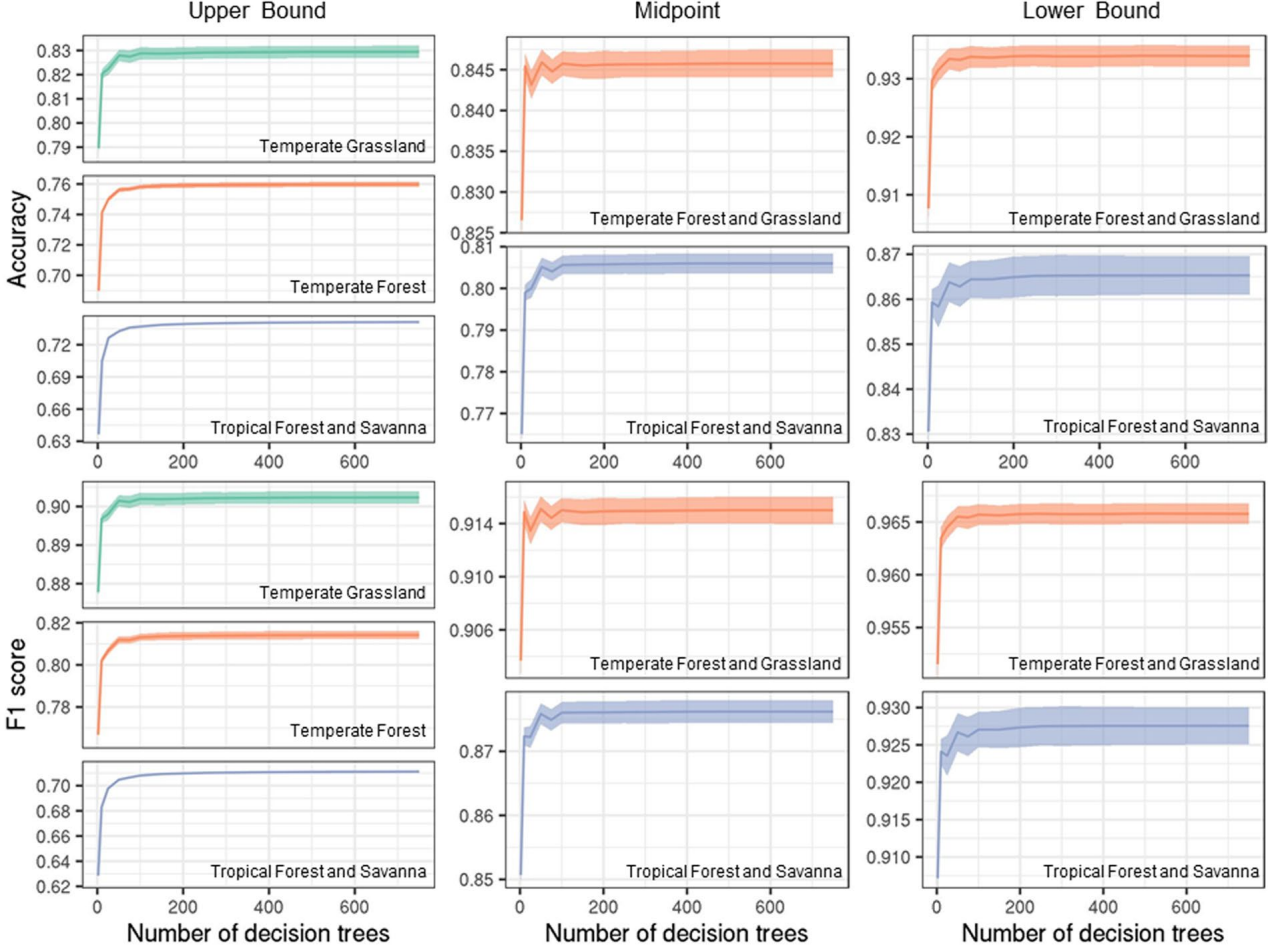
Performance of random forest models in terms of classification accuracy and F1 score with different numbers of decision trees. For each biome (Fig. 2e), we tested a different number of decision trees in the random forest ranging from 2 to 750. The solid lines represent the mean of 10 repetitions, and the bands represent the standard deviation. The number of trees = 100 for the upper bound and midpoint models and 200 for the lower bound model were chosen to maximize the model performance while minimizing computational time.

**Fig. 6:**
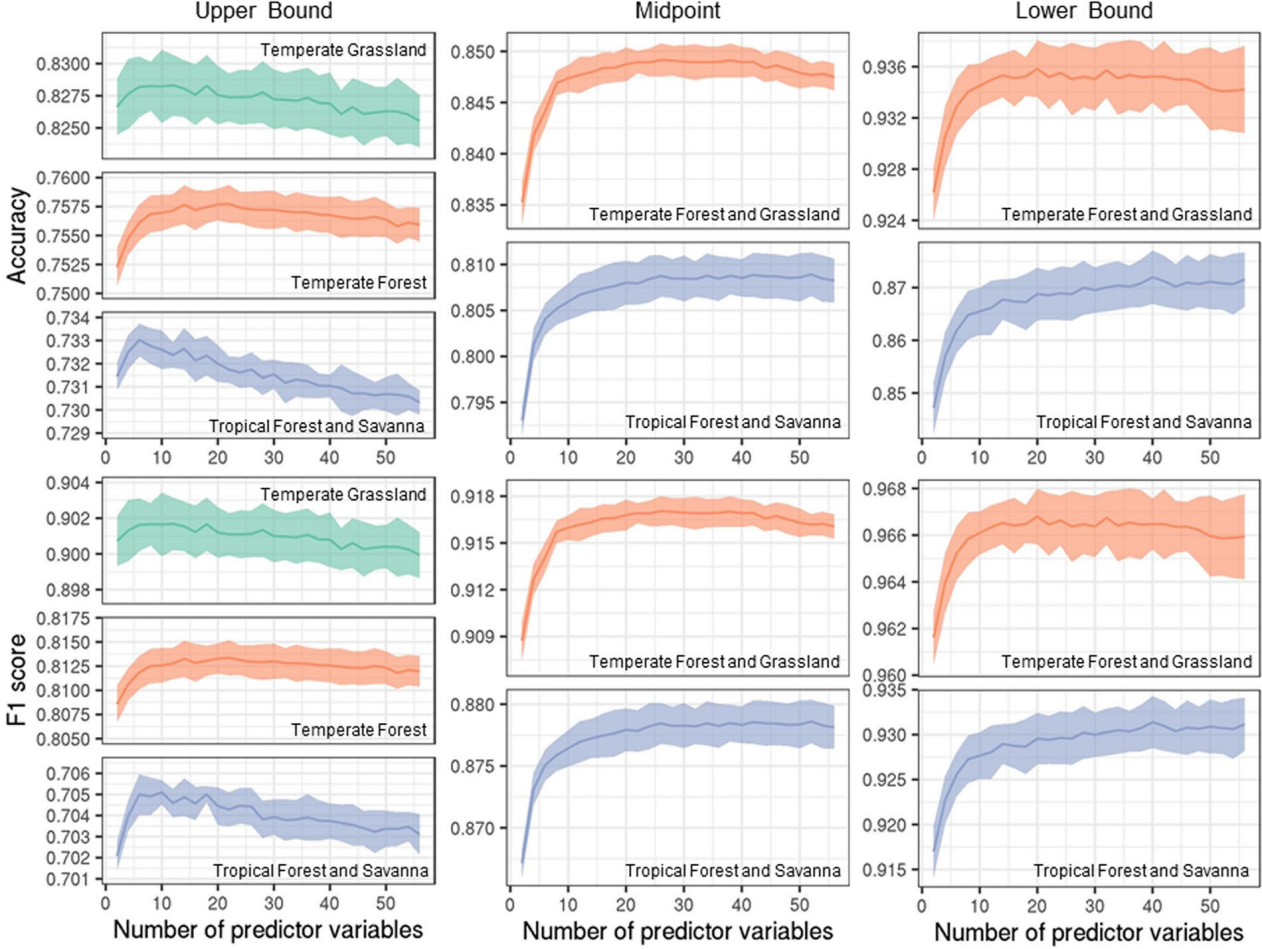
Performance of random forest models in terms of classification accuracy and F1 score with different numbers of predictor variables. For each biome (Fig. 2e), we tested a different number of predictor variables in the random forest ranging from 2 to 56. The solid lines represent the mean of 10 repetitions, and the bands represent the standard deviation. We used the default number of predictor variables (seven) for all biomes for the upper bound model. We chose 26 and 42 for Temperate Forest and Grassland and Tropical Forest and Savanna for the midpoint model. We chose 20 and 40 for Temperate Forest and Grassland and Tropical Forest and Savanna for the lower bound model.

To improve the performance of the model while minimizing the time it takes to compute, we adjusted two hyperparameters of the RF algorithm: the number of decision trees and the number of predictor variables. Similar to the cross-validation described above, we randomly sampled 50,000 points (25,000 for each class) for the upper bound and midpoint models and 80% of the sample points for the lower bound model for each of the ten repetitions to assess RF performance using different hyperparameter values (Table 1). Specifically, we calculated the classification accuracy and F1 score for different hyperparameter values. Based on 10 iterations, we chose the number of 100 decision trees for the upper bound and midpoint models and 200 for the lower bound model where both accuracy and F1 score converged (Fig. 5). We used the default number of predictor variables (seven) for all biomes for the upper bound model. We chose 26 and 42 for Temperate Forest and Grassland and Tropical Forest and Savanna, respectively, for the midpoint model (Fig. 6). We chose 20 and 40 for Temperate Forest and Grassland and Tropical Forest and Savanna, respectively, for the lower bound model (Fig. 6).

**Fig. 7:**
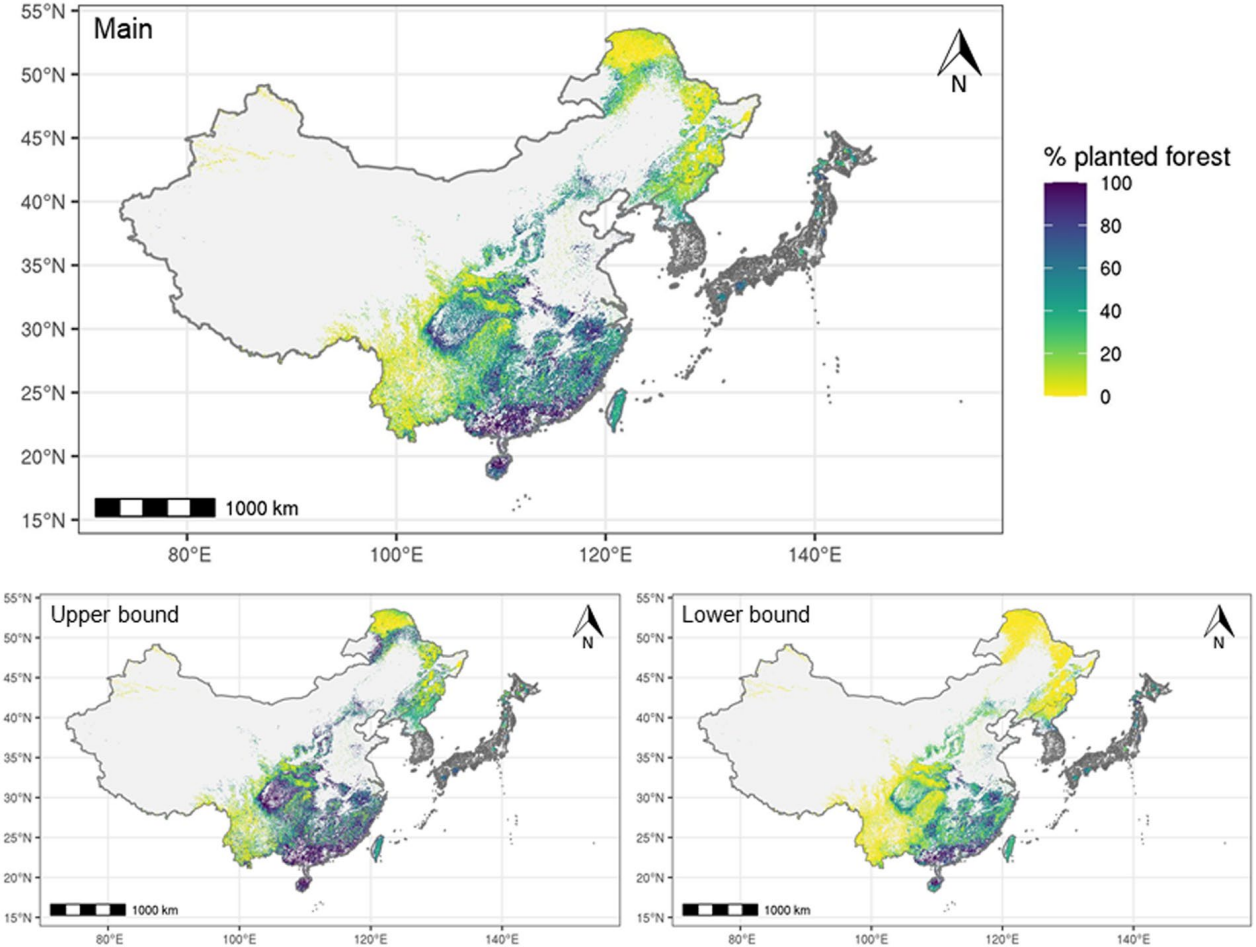
Spatial distribution of percent planted forest in East Asia. Our main prediction was the mean percent planted forest from the three models (upper bound, midpoint, and lower bound), while upper and lower bounds present potential ranges. Prediction was made for China, DPRK, and small portions of Japan. National planted forest maps of Japan^12^ and ROK^16^ were used for the remaining areas in ROK and the majority of areas in Japan, indicated in gray. The data is in a vector format with each polygon representing a 0.0090° by 0.0090° (approximately 1 km) grid in the WGS84 datum.

**Fig. 8:**
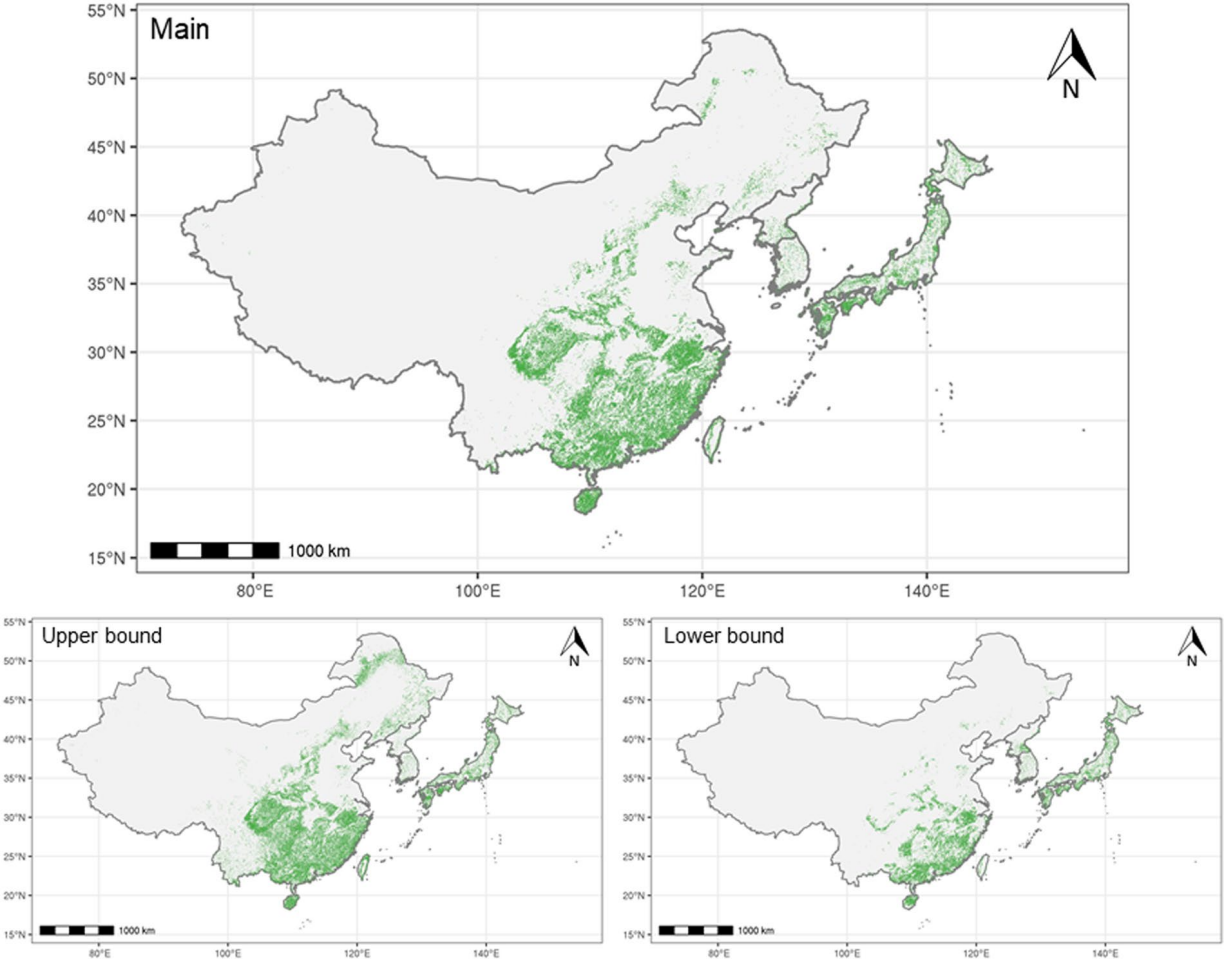
Spatial distribution of planted forests in East Asia. The map shows the estimated areas where the percent planted forest is greater than 50%. For ROK and most areas in Japan, national planted forest maps^12,16^ were used to determine the distribution of planted forest. The data is in a vector format with each polygon representing a 0.0090° by 0.0090° (approximately 1 km) grid in the WGS84 datum.

For the final RF model, we ensured that the training set had an equal number of points for each class (*i.e*., 50% planted forest and 50% natural forest) by randomly under-sampling the dominant class. The prediction of our classification model was the percent planted forest based on how many decision trees returned the “planted” prediction. We built 20 models to derive the mean percentage for each biome and model (upper bound, midpoint, and lower bound) (Table 1). Finally, we calculated the mean percentage of the three models as a final value, while upper and lower bounds serve as a potential range (Fig. 7). Grid cells with a predicted percentage ≥50% are considered planted forest (Fig. 8). Using the spatially continuous dataset of 57 predictor variables (see *Data fusion*), we created a map covering the entire forested area in East Asia using model prediction.

### Mapping dominant tree species of the planted forests

**Fig. 9:**
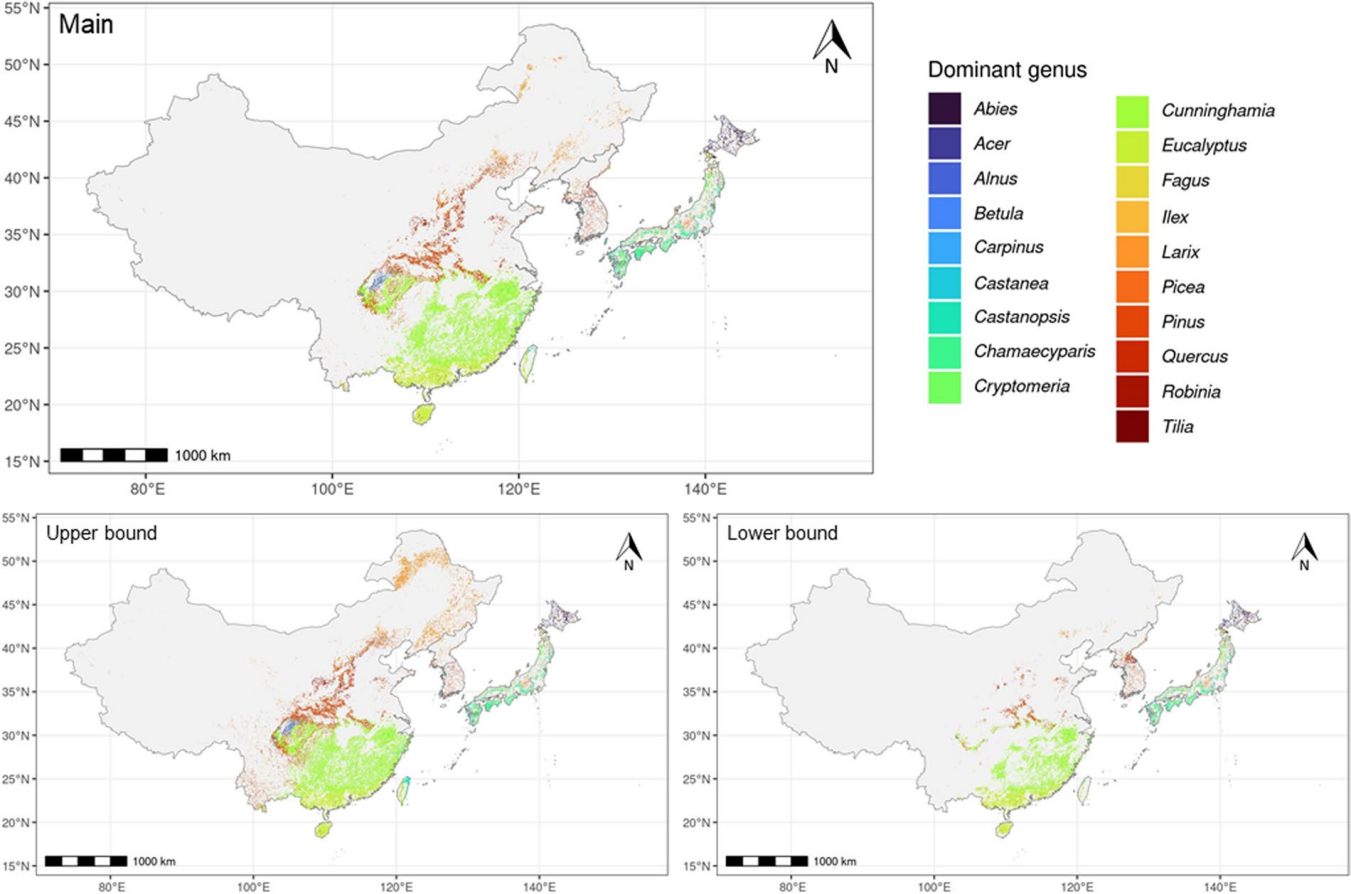
Spatial distribution of dominant tree species to the genus level across the planted forest range in East Asia (Fig. 8).

Over the planted forest expanse in East Asia identified by the final RF classification model, we predicted the dominant tree species (to the genus level) of the planted forest for each criterion (Fig. 9). For the training set, we combined 2,481 *in situ* records in China^20–265^ with the tree-level records of Japan^295^ and ROK^296^ National Forest Inventories (NFI). Specifically, we calculated importance value for each species for each NFI plot within the predicted planted forest expanse and identified the species with the highest importance value as the dominant species for the given plot. Importance value is the sum of the percent basal area and the percent number of individuals of each species and represents the overall dominance of the species^297,298^. After identifying the dominant species for each NFI plot, we aggregated the plots into the 0.009° by 0.009° grid cells by taking the majority vote of the dominant species. We retained the genus names of the dominant species, and only genera with 60 or more samples were included to ensure a sufficient size of training data.

We trained an RF classification model using the same package in R, with the default hyperparameter setting and an identical set of predictor variables, except for roadless areas and GEDI attributes due to a substantial number of missing values (86% and 34% of the sample size, respectively). We ensured that the training set had an equal number of points for each class (*i.e*., genus) by combining random under-sampling and oversampling using the “UBL” package in R^299^. To assess the performance of the RF model in mapping dominant genera across the planted forest expanse in East Asia, we performed a 90/10 cross-validation using bootstrapping. In each iteration, we used stratified sampling to split the entire training dataset into the training (90%) and testing (10%) sets using the “caret” package in R^300^ and conducted a combination of under-sampling and oversampling of the training set to address the class imbalance (Table 1). Based on 100 random iterations, we calculated the 95% CI of overall classification accuracy and precision, recall, and F1 score for each class.

## Data Records

The spatial database of planted forests consists of maps of estimated planted forest distribution (Figs. 7, 8) and dominant tree species (Fig. 9) of East Asia, available at 10.6084/m9.figshare.21774725.v3^301^. The database is in shapefiles where each polygon is 0.009° by 0.009° in size within the forested area of 2020 (≥5 m tree height) based on the FAO’s definition of “forest”^2,270^. Each polygon contains the following attributes:

**ID:** Polygon ID

**Biome:** Biome classes used in the study

**Country:** Country

**Prc_Pln:** Percent planted forest. The values represented the average of the three models (upper bound, midpoint, and lower bound). NA for ROK and a majority of areas in Japan, where national planted forest maps^12,16^ were used as the final planted/natural label (Fig. 2f).

**Prc_P_U:** Percent planted forest predicted by the upper bound model. NA for ROK and a majority of areas in Japan, where national planted forest maps^12,16^ were used as the final planted/natural label (Fig. 2f). Note that values are not always higher than Prc_Pln.

**Prc_P_L:** Percent planted forest predicted by the lower bound model. NA for ROK and a majority of areas in Japan, where national planted forest maps^12,16^ were used as the final planted/natural label (Fig. 2f). Note that values are not always lower than Prc_Pln.

**Type:** “Planted” or “Natural” forests based on the main result (*i.e*., the average of the three models). For our predicted percent planted forest, “Planted” if Prc_Pln ≥ 0.5 and “Natural” if Prc_Pln < 0.5. For Prc_Pln = NA, national planted forest maps^12,16^ were used to determine if the given polygon is a planted forest, and if not, “Natural.”

**Typ_Upp:** “Planted” or “Natural” forests based on the upper-bound model.

**Typ_Lwr:** “Planted” or “Natural” forests based on the lower-bound model.

**Genus:** For Type = “Planted”, this attribute indicates the predicted dominant genus. NA for Type = “Natural”.

**Gns_Upp:** For Typ_Upp = “Planted”, this attribute indicates the predicted dominant genus. NA for Typ_Upp = “Natural”.

**Gns_Lwr:** For Typ_Lwr = “Planted”, this attribute indicates the predicted dominant genus. NA for Typ_Lwr = “Natural”.

**Besnard_Yr:** Estimated planted year based on forest age^302^ (10.17871/ForestAgeBGI.2021). See Usage Notes.

**Du_Yr:** Estimated planted year based on the map of planting year of plantations^303,304^ (10.6084/m9.figshare.19070084.v2). A value of 1981 indicates the planting year was before 1982, and values from 1982 to 2019 correspond to the planting years. See Usage Notes.

**Area_m2:** Area of the planted forest polygons in square meters.

Raster layers are also available for percent planted forest, type (planted or natural forest), and dominant genus, at 10.6084/m9.figshare.21774725.v3^301^.

Based on our prediction, the total area of planted forests in East Asia was 948,863 km^2^, ranging between 600,529 and 1,277,549 km^2^. China shared 87% of the planted forest area in East Asia, most of which is in the lowland subtropical and tropical regions, and Sichuan Basin (Fig. 8). More than half of China’s planted forest area was dominated by *Cunninghamia* (Table 2) in the subtropical region and Sichuan Basin (Fig. 9). Larch (*Larix* spp.), black locust (*Robinia* spp.), and pine (*Pinus* spp.) were widely observed in northern and central China, and eucalyptus dominated planted forests in tropical regions.

**Table 2:** Predicted area of planted forest for each dominant genus based on the main model.

Genus	Predicated area in km^2^ (%)				
	East Asia	China	Japan	ROK	DPRK
*Cunninghamia*	480,913 (50.7)	480,901 (58.2)	12 (0.0)	0 (0.0)	0 (0.0)
*Pinus*	151,749 (16.0)	142,031 (17.2)	2,334 (2.2)	5,692 (60.0)	1,691 (21.2)
*Eucalyptus*	137,318 (14.5)	137,318 (16.6)	0 (0.0)	0 (0.0)	0 (0.0)
*Larix*	40,487 (4.3)	32,400 (3.9)	5,383 (5.1)	525 (5.5)	2,179 (27.3)
*Chamaecyparis*	35,267 (3.7)	3,117 (0.4)	32,115 (30.4)	35 (0.4)	0 (0.0)
*Cryptomeria*	22,772 (2.4)	798 (0.1)	21,973 (20.8)	1 (0.0)	0 (0.0)
*Robinia*	14,046 (1.5)	13,858 (1.7)	87 (0.1)	100 (1.1)	1 (0.0)
*Abies*	11,695 (1.2)	211 (0.0)	11,455 (10.8)	0 (0.0)	29 (0.4)
*Alnus*	11,140 (1.2)	10,438 (1.3)	697 (0.7)	4 (0.0)	0 (0.0)
*Quercus*	10,793 (1.1)	21 (0.0)	4,510 (4.3)	2,402 (25.3)	3,860 (48.3)
*Castanopsis*	10,367 (1.1)	3,032 (0.4)	7,333 (6.9)	2 (0.0)	0 (0.0)
*Fagus*	5,892 (0.6)	4 (0.0)	5,887 (5.6)	0 (0.0)	0 (0.0)
*Castanea*	3,669 (0.4)	0 (0.0)	3,089 (2.9)	561 (5.9)	19 (0.2)
*Carpinus*	3,530 (0.4)	437 (0.1)	2,748 (2.6)	143 (1.5)	203 (2.5)
*Ilex*	3,515 (0.4)	8 (0.0)	3,507 (3.3)	0 (0.0)	0 (0.0)
*Acer*	2,640 (0.3)	1 (0.0)	2,637 (2.5)	1 (0.0)	2 (0.0)
*Betula*	1,979 (0.2)	885 (0.1)	1,092 (1.0)	2 (0.0)	0 (0.0)
*Picea*	681 (0.1)	289 (0.0)	392 (0.4)	0 (0.0)	0 (0.0)
*Tilia*	410 (0.0)	0 (0.0)	381 (0.4)	26 (0.3)	3 (0.0)
Total	948,863 (100.0)	825,751 (100.0)	105,633 (100.0)	9,493 (100.0)	7,986 (100.0)

East Asia is the sum of all four countries. The numbers in parenthesis represent percent area in each country or region.

In Japan and ROK, planted forests were uniformly distributed across the country (Fig. 8). More than half of Japan’s total planted forest area was *Chamaecyparis*- or *Cryptomeria*-dominant (Table 2), while other coniferous genera (*e.g*., *Abies* and *Pinus*) covered northern planted forests (Fig. 9). ROK’s planted forests were characterized by diverse genera; more than half of planted forest areas were dominated by pine, followed by deciduous trees including oak (*Quercus* spp.) and chestnut (*Castanea* spp.). DPRK’s planted forests were mainly distributed in the south, largely composed of oak, larch, and pine.

The input training data, including the response variable and predictor variables, used in this study are available at 10.6084/m9.figshare.21774812.v2^305^. Underlying data included *in situ* and digitized planted-natural forest data:

**The** ***in situ*** **observational data of China**^20–265^

**The Japan Vegetation Map**^12^ (http://gis.biodic.go.jp/webgis/sc-025.html?kind=vg67)

**The national planted forest map of China** ^15^

**The national planted forest map of ROK** ^16^

**SDPT version 1.0**^13^ (https://www.wri.org/research/spatial-database-planted-trees-sdpt-version-10)

**Global planted trees extent 2015**^14^ (10.5281/zenodo.3931930)

**Japan National Forest Inventory**^295^ (http://forestbio.jp/datafile/datafile.html)

**ROK National Forest Inventory** ^296^

The predictor variables used in this study are all available through open sources as follows:

**GEDI L2B**^269^ (https://search.earthdata.nasa.gov/search)

**Tree height** (https://glad.umd.edu/dataset/GLCLUC2020)

**MODIS** (https://modis.gsfc.nasa.gov/)

**Corrected precipitation**: PBCOR^271^ (http://www.gloh2o.org/pbcor/)

**Bioclimate data:** CHELSA^272,273^ (https://chelsa-climate.org/bioclim/)

**Global aridity index and potential evapotranspiration**: CGIAR-CSI v.2^274^ (10.6084/m9.figshare.7504448.v3)

**Topography**: EarthEnv^275^ (http://www.earthenv.org/topography)

**Global cattle distribution**^276^ (10.7910/DVN/GIVQ75)

**Roadless area**^277^ (10.1126/science.aaf7166)

**Protected area**: UNEP-WCMC^278^ (https://www.protectedplanet.net/en)

**Human footprint**^279^ (10.5061/dryad.052q5)

**Soil characteristics**: WISE30sec v1.0^280^ (https://www.isric.org/explore/wise-databases)

Other data used in this study include:

**The Nature Conservancy (TNC) Terrestrial Ecoregions map**^19^ (https://geospatial.tnc.org/datasets/b1636d640ede4d6ca8f5e369f2dc368b/about)

All the data listed above are open access, except **the national planted forest map of China**^15^, t**he national planted forest map of ROK**^16^, and **the ROK National Forest Inventory**^296^. The sensitive information in these datasets will be available upon request via Science-i (https://science-i.org/) and approval from data contributors.

## Technical Validation

### Model validation in imputing GEDI missing values

We conducted cross-validation with bootstrapping to evaluate the model in imputing the missing values in GEDI attributes for the high-latitude areas (Supplementary Table S1; see *Quality-Oriented Data Integration (QODI)* in Methods). R^2^ was within the range of 31% and 42% for all the GEDI attributes in Temperate Grassland and Temperate Forest (Table 3). For Tropical Forest and Savanna, canopy height showed R^2^ of 22%, and the rest of the attributes showed R^2^ of almost 30%. Foliage height diversity showed the highest R^2^ and total canopy cover showed the lowest root mean square error (RMSE) among all GEDI attributes in all groups (Table 3).

**Table 3:** Evaluation in imputing missing data of GEDI attributes for mapping purposes.

Biome	Response variable (unit)	RMSE (mean±95%CI)	R^2^ (mean±95%CI)
Temperate Grassland	Canopy height (rh100) (cm)	560.80 ± 1.57	0.35308 ± 0.00226
	Plant area index (pai) (-)	0.73181 ± 0.00093	0.38590 ± 0.00158
	Foliage height diversity (fhd_normal) (-)	0.29674 ± 0.00058	0.42148 ± 0.00167
	Total canopy cover (cover) (%)	0.15642 ± 0.00018	0.39395 ± 0.00166
Temperate Forest	Canopy height (rh100) (cm)	614.98 ± 0.21	0.31465 ± 0.00021
	Plant area index (pai) (-)	0.88935 ± 0.00016	0.39003 ± 0.00013
	Foliage height diversity (fhd_normal) (-)	0.29609 ± 0.00006	0.41618 ± 0.00012
	Total canopy cover (cover) (%)	0.16938 ± 0.00002	0.39714 ± 0.00012
Tropical Forest and Savanna	Canopy height (rh100) (cm)	716.28 ± 0.38	0.22118 ± 0.00025
	Plant area index (pai) (-)	1.01696 ± 0.00032	0.28806 ± 0.00017
	Foliage height diversity (fhd_normal) (-)	0.30687 ± 0.00009	0.29766 ± 0.00024
	Total canopy cover (cover) (%)	0.16035 ± 0.00004	0.29317 ± 0.00018

We conducted cross-validation with bootstrapping. The mean and 95% confidence interval (CI) from 20 iterations are shown for root mean square error (RMSE) and R-squared (R^2^).

### Model validation in estimating planted forests

To evaluate the performance of our mapping product of East Asia, we compared our main prediction (Fig. 8) with the planted/natural labels of the midpoint dataset for China. We calculated classification accuracy, precision, recall, F1 score, and four elements of confusion matrices in percentage (true positive, false positive, false negative, and true negative, where positive class represented planted, and negative class represented natural forest). Our prediction is characterized by a high recall (0.99), indicating that 99% of the observed planted forests were correctly predicted as planted forest (Table 4). Our precision was 0.63, which indicates that approximately two out of three positive predictions are actually planted forests. This level of accuracy is similar to those of other large-scale forest mapping studies (0.60–0.80)^306–308^.

**Table 4:** Evaluation metrics and elements of confusion matrices of the main prediction of planted forest distribution.

Evaluation metrics	Value
Accuracy	0.945
Precision	0.633
Recall	0.990
F1 score	0.772
True positive	0.093
False positive	0.054
False negative	0.001
True negative	0.853

Our final prediction was evaluated against the planted/natural labels of the midpoint dataset in China. The elements of confusion matrices are represented in percentages. The positive class represents planted, and the negative class represents natural forests. Accuracy shows the proportion of overall correct prediction, precision represents the correct prediction of the positive class (*i.e*., planted) among all positive predictions, recall represents the correct prediction of the positive class among all actual positive cases, and F1 score represents a balanced score of precision and recall.

While precision is often negatively associated with recall, the F1 score, 0.77, indicates that our model is well-balanced between precision and recall. The low precision is attributable to the imbalanced distribution of positive and negative classes in the validation set (the midpoint dataset for China). The number of samples for natural forests was almost 10 times greater than that of planted forests in our validation set (Table 4). While we maximized the predictive performance by balancing the training data, high accuracy and low precision are inevitable due to the imbalanced validation set.

To further validate the quality of our prediction, we also compared our estimated total area of planted forests against the reported values from the FAO Global Forest Resources Assessment (FRA)^2^ and the National Forest Inventory dataset from China^309^ (Table 5). Our total predicted area of planted forests in East Asia was 948,863 km^2^ with a range between 600,529 and 1,277,549 km^2^, which is consistent with the FRA estimate (981,390 km^2^). The predicted area of China’s planted forests was 825,751 km^2^ (475,566–1,159,009 km^2^), while the FRA reports 846,960 km^2^ and the Ninth National Forest Inventory of China reports 795,428 km^2^. For Japan, the range of estimated areas of planted forests was between 103,447 and 105,633 km^2^, while the FRA reported value is 101,840 km^2^. Our estimated area of planted forests in DPRK was 7,986 km^2^ (5,601–11,648 km^2^), while the FRA reported value is 9,870 km^2^. Overall, our estimate was consistent with those reported by the FRA and the National Forest Inventory of China.

**Table 5:** Predicted area of planted forest for each country and the entire region and estimated area by other sources.

Country or region	Predicted area main (km^2^)	Predicted area upper bound model (km^2^)	Predicted area lower bound model (km^2^)	FAO’s FRA 2020 (km^2^)^2^	The Ninth National Forest Inventory of China (km^2^)^309^
East Asia	948,863	1,277,549	600,529	981,390	NA
China	825,751	1,159,009	475,566	846,960	795,428
Japan	105,633	103,447	103,823	101,840	NA
DPRK	7,986	5,601	11,648	9,870	NA

ROK is not shown here as the national planted forest map^16^ was used in the final map. East Asia includes China, Japan, ROK, and DPRK.

### Model validation in estimating dominant tree species

Our 90/10 bootstrapping cross-validation in estimating the dominant tree species across planted forests showed an overall classification accuracy of 0.396 (±0.003 95% CI). Among all the planted tree species, *Cunninghamia* and *Eucalyptus* had the highest F1 score (0.745 and 0.733, respectively), with high recall (0.893 and 0.802, respectively) and satisfactory precision (0.644 and 0.403, respectively) (Table 6). Meanwhile, *Carpinus* and *Castanea* showed the lowest F1 score (0.124 and 0.136, respectively), which likely resulted from a small sample size compared to other genera. *Acer*, *Alnus*, *Betula*, *Cryptomeria*, *Picea*, *Pinus*, *Quercus*, and *Tilia* showed low recall compared to precision, indicating that true labels for these genera tended to be classified as other genera. *Abies*, *Carpinus*, *Castanea*, *Castanopsis*, *Chamaecyparis*, *Cunninghamia*, *Eucalyptus*, *Fagus*, *Ilex*, *Larix*, and *Robinia* had lower precision than recall due to the overprediction of these genera (Table 6).

**Table 6:** Evaluation of the random forest classification model in mapping the dominant tree species across the planted forest expanse in East Asia.

Genus	Precision (mean ± 95%CI)	Recall (mean ± 95%CI)	F1 score (mean ± 95%CI)
*Abies*	0.393 ± 0.007	0.679 ± 0.013	0.497 ± 0.008
*Acer*	0.203 ± 0.018	0.125 ± 0.012	0.154 ± 0.013
*Alnus*	0.039 ± 0.028	0.011 ± 0.007	0.182 ± 0.005
*Betula*	0.288 ± 0.020	0.187 ± 0.014	0.223 ± 0.015
*Carpinus*	0.086 ± 0.013	0.129 ± 0.018	0.124 ± 0.011
*Castanea*	0.078 ± 0.012	0.152 ± 0.023	0.136 ± 0.012
*Castanopsis*	0.163 ± 0.008	0.522 ± 0.027	0.246 ± 0.011
*Chamaecyparis*	0.404 ± 0.007	0.525 ± 0.012	0.456 ± 0.008
*Cryptomeria*	0.541 ± 0.008	0.352 ± 0.008	0.425 ± 0.007
*Cunninghamia*	0.644 ± 0.018	0.893 ± 0.014	0.745 ± 0.014
*Eucalyptus*	0.694 ± 0.030	0.802 ± 0.030	0.733 ± 0.024
*Fagus*	0.403 ± 0.008	0.771 ± 0.015	0.527 ± 0.009
*Ilex*	0.131 ± 0.011	0.436 ± 0.034	0.200 ± 0.016
*Larix*	0.437 ± 0.011	0.502 ± 0.014	0.465 ± 0.011
*Picea*	0.265 ± 0.043	0.190 ± 0.031	0.282 ± 0.024
*Pinus*	0.584 ± 0.010	0.445 ± 0.010	0.504 ± 0.009
*Quercus*	0.389 ± 0.013	0.151 ± 0.006	0.216 ± 0.008
*Robinia*	0.460 ± 0.035	0.527 ± 0.037	0.479 ± 0.029
*Tilia*	0.057 ± 0.024	0.031 ± 0.012	0.168 ± 0.010

We conducted a rigorous 90/10 bootstrapping cross-validation. The mean and 95% confidence interval (CI) are shown for the precision, recall, and F1 score of each class (*i.e*., genus) based on 100 random iterations.

### Uncertainties

While this study advances the current understanding of planted forests in East Asia based on multi-source data consisting of *in situ*, digitized, and modeled datasets, uncertainties arose from two main sources. First, limited *in situ* data, especially from Japan, ROK, and DPRK constitute one of the largest sources of uncertainties. The limited *in situ* data from these countries could lead to lower accuracy in our planted forests prediction. Nevertheless, to mitigate this uncertainty, we integrated different data sources for modeling (*e.g*., SDPT^13^ and the Global Planted Trees Extent 2015^14^), and the final map product for these countries relied on external sources^12,16^.

Secondly, our map of planted tree species depicts the spatial distribution of the dominant tree species to the genus level across the range of planted forests. However, it is beyond the scope of this study to identify the spatial distribution of monoculture planted forests versus mixed-species planted forests, the latter of which are common in certain regions^310^. This uncertainty in tree species richness can be mitigated by integrating the mapping products presented here with recent global high-resolution maps of local tree species richness and co-limitation^289^. Furthermore, some genera predicted in our study had low F1 scores, which can be mitigated by increasing the sample size for these species. Nevertheless, it is not realistic to achieve perfectly balanced data, and differences in predictive performance among genera are inevitable.

## Usage Notes

Our final maps of planted forest range (Fig. 8) for Japan and ROK consist of data directly obtained from the national planted forest maps of Japan^12^ and ROK^16^. Users of these particular maps should cite these sources accordingly.

Planted forests in this study include forests of all ages that have been planted for ecological restoration, commercial plantation, and other purposes, such as landscape and disaster prevention.

**Fig. 10:**
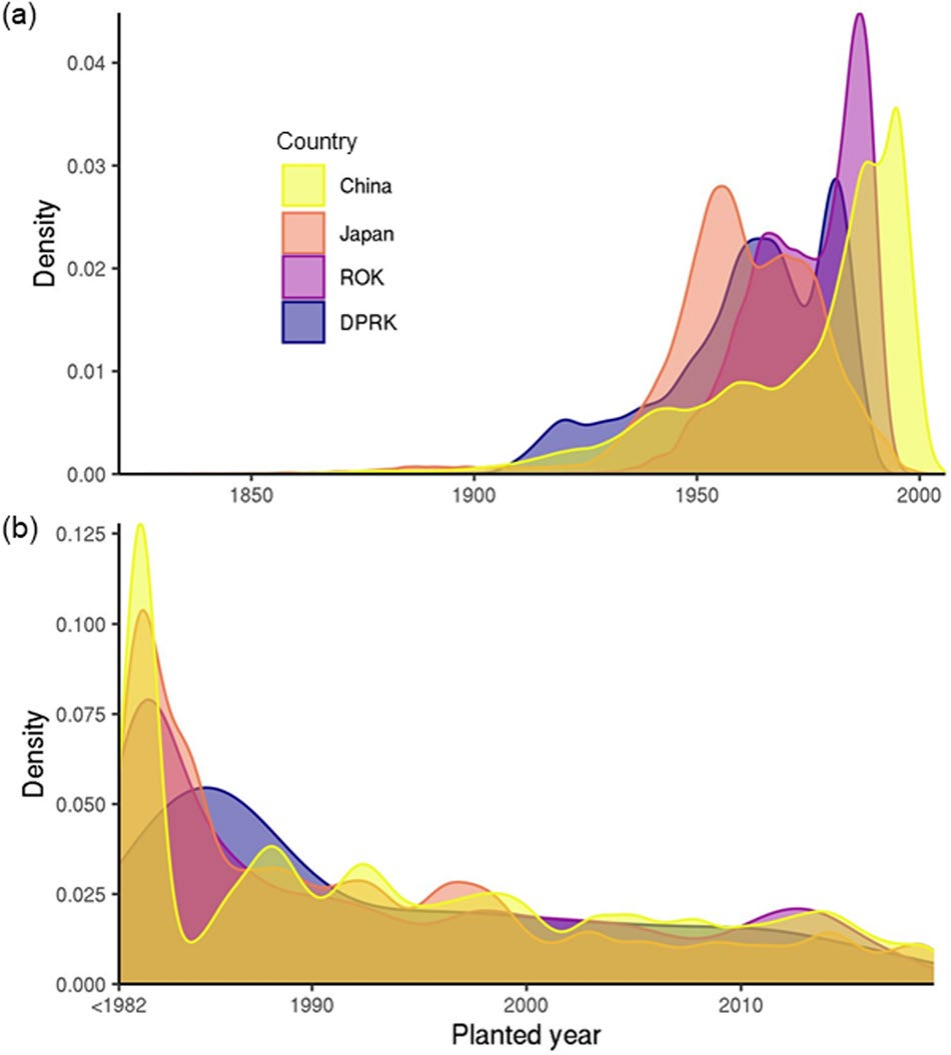
Density plot showing the concentration of estimated planted forests in the range of planted year for each country. (**a**) planted year was estimated based on forest age^302^ with a maximum value of 2010. (**b**) planted year was estimated based on the map of planting year of plantations^303,304^.

Since the underlying training datasets differ by planting years, we were only able to quantify a roughly estimated range of underlying years. Specifically, we overlaid our final map with two existing map layers with estimated forest age^302^ and planted year^303,304^ values. Based on these two sources, some planted forests were planted more than 100 years ago, while other planted forests are less than five years in age (Fig. 10). Estimation based on forest age^302^ presents consistency with planting history in each country; the majority of planted forests were established post-war in Japan, followed by efforts in the Korean peninsula, while planted forests in China come from more recent planting (Fig. 10a). We included planted year information in our map product (see Data Records).

## Supplementary information

Supplementary Table S1

## Data Availability

The R code, saved RF models, and training datasets to reproduce the results of this study are available at 10.6084/m9.figshare.21774812.v2^305^.
